# Crystal structure of bis­[(η^5^-*tert*-butyl­cyclo­pentadien­yl)tri­carbonyl­molybdenum(I)](*Mo*—*Mo*)

**DOI:** 10.1107/S2056989024006959

**Published:** 2024-07-23

**Authors:** Nigar Z. Ibrahimova, Dilgam B. Tagiyev, Iltifat U. Lyatifov, Mehmet Akkurt, Khudayar I. Hasanov, Ajaya Bhattarai

**Affiliations:** a Acad. M. Nagiev Institute of Catalysis and Inorganic Chemistry, Ministry of Science and Education of the Azerbaijan Republic, Azerbaijan; b Department of Physics, Faculty of Sciences, Erciyes University, 38039 Kayseri, Türkiye; c Western Caspian University, Istiqlaliyyat Street 31, AZ 1001, Baku, Azerbaijan; d Azerbaijan Medical University, Scientific Research Centre (SRC), A. Kasumzade St. 14. AZ 1022, Baku, Azerbaijan; e Department of Chemistry, M.M.A.M.C (Tribhuvan University) Biratnagar, Nepal; Vienna University of Technology, Austria

**Keywords:** crystal structure, Mo complex, carbonyl ligands, cyclo­penta­dienyl ligand, tert-butyl group, carbonyl group, binuclear complex, steric effect

## Abstract

The dinuclear mol­ecule of the title compound, [Mo_2_(C_9_H_13_)_2_(CO)_6_] or [Mo(^*t*^BuCp)(CO)_3_]_2_ where ^*t*^Bu and Cp are *tert*-butyl and cyclo­penta­dienyl, is centrosymmetric and is characterized by an Mo—Mo bond length of 3.2323 (3) Å. Imposed by inversion symmetry, the ^*t*^BuCp and the carbonyl ligands are in a *transoid* arrangement to each other. In the crystal, inter­molecular C—H⋯O contacts lead to the formation of layers parallel to the *bc* plane.

## Abstract 2

The Mo—Mo bond in the dinuclear mol­ecular title compound is 3.2323 (3) Å, in good agreement with related dinuclear molybdenum(I) compounds with cyclo­penta­dienyl (Cp) ligands.

## 1. Chemical context

Cyclo­penta­dienyl (Cp) complexes can be employed as versatile precursors for the synthesis of new functional materials, including heterocycles, catalysts, organic conductors or pharmaceuticals (Absolonová *et al.*, 2021 ▸; Kharitonov *et al.*, 2022 ▸). Not only the exchange of the central metal atoms to which the Cp ligands are bound, but also the decoration of Cp ligands with functional groups can be used as a synthetic strategy to develop new catalysts (Loginov *et al.*, 2019 ▸). Similarly to other coordination compounds (Mahmoudi *et al.*, 2017*a* ▸,*b* ▸; Mahmudov & Pombeiro 2023 ▸), the inter­play between electron-donating or -withdrawing functions of substituents with their non-covalent donor or acceptor character in cyclo­penta­dienyl complexes can improve activity and selectivity of catalytic transformations.
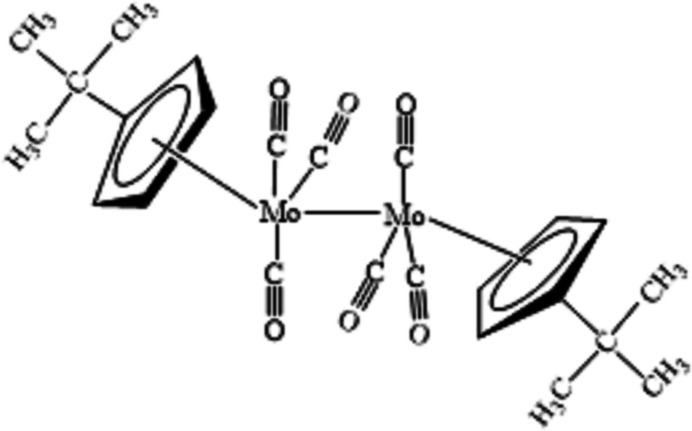


In this context, we report the synthesis and crystal structure analysis of a dinuclear bis­(*tert*-butyl­cyclo­penta­dien­yl)hexa­carbonyl­dimolybdenum(I) complex, [Mo(C_9_H_13_)(CO)_3_]_2_ or [Mo(^*t*^BuCp)(CO)_3_]_2_ (^*t*^Bu and Cp represent *tert*-butyl and cyclo­penta­dien­yl).

## 2. Structural commentary

**Figure 1:**
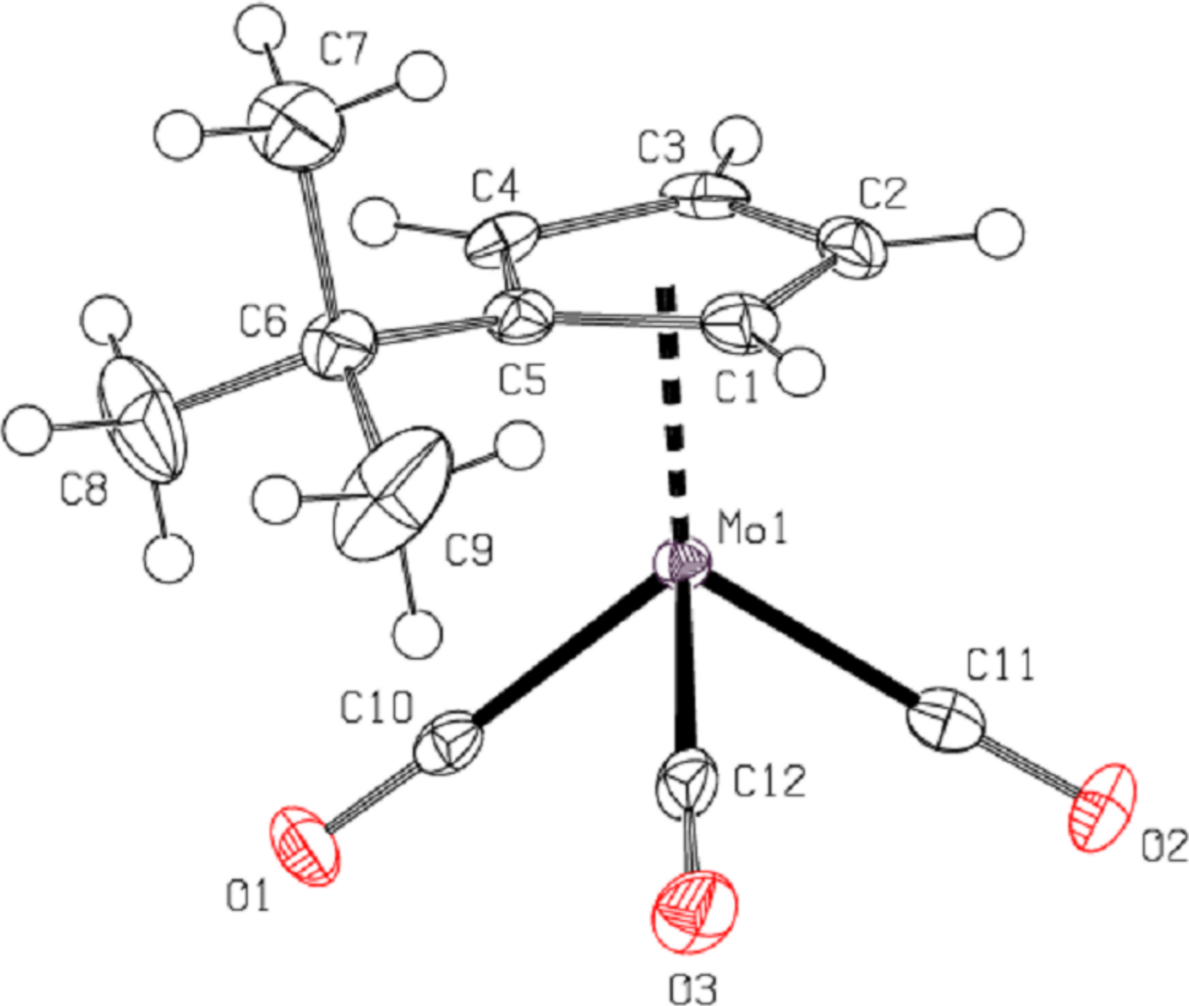
The asymmetric unit of the title compound, showing the labeling scheme. Displacement ellipsoids are shown at the 50% probability level.

**Figure 2:**
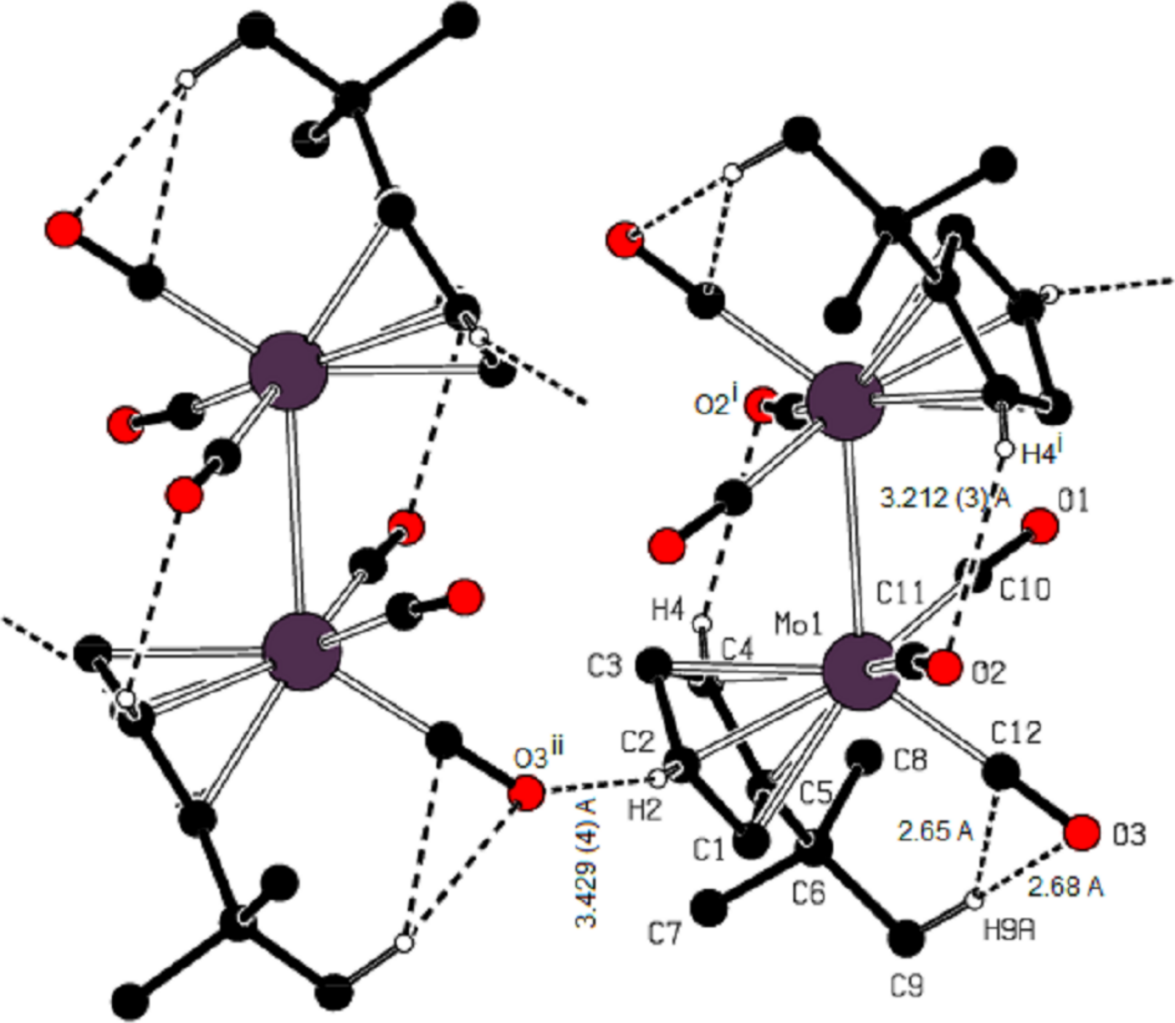
The intra- and inter­molecular hydrogen contacts within the crystal structure of the title compound. [Symmetry codes: (i) −*x* + 1, −*y* + 1, −*z* + 1; (ii) *x*, −*y* + 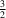, *z* + 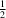.]

The dinuclear title complex crystallizes in the monoclinic space group *P*2_1_/*c* with half of the mol­ecule present in the asymmetric unit (Fig. 1 ▸). The entire dimer is generated by an inversion center located at the middle of the Mo—Mo bond (Fig. 2 ▸). Imposed by inversion symmetry, the *tert*-butyl­cyclo­penta­dienyl (^*t*^BuCp) ligands are in a transoid arrangement about the Mo—Mo bond with that bond being 3.2323 (3) Å in length. For the *tert*-butyl groups on the (^*t*^BuCp) ring, the Mo1^i^—Mo1—C5—C6 torsion angle is 115.30 (18)°. All bond angles involving the carbonyl ligands are close to linearity, with Mo1—C10≡O1, Mo1—C11≡O2 and Mo1—C12≡O3 being 174.6 (2), 173.1 (2) and 178.2 (2)°, respectively.

The mol­ecule of the title compound is sterically strained, which seems to be caused by short non-valent CO⋯C^i^O^i^ and ^*t*^BuCp⋯C^ii^O^ii^ contacts, as well as steric inter­action between the *tert*-butyl and C12≡O3 groups [symmetry codes: (i) −*x* + 1, −*y* + 1, −*z* + 1; (ii) *x*, −*y* + 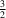, *z* + 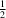]. The C12⋯H9*A* and O3⋯H9*A* contacts have values as small as 2.65 and 2.68 Å, respectively (Fig. 2 ▸). The torsion angles C1—C5—C6—C7 and C4—C5—C6—C7 are 87.0 (3)° and −80.5 (3)°, respectively. The steric influence of the *tert*-butyl group is also evident in the –Mo(CO)_3_– fragment with a C12—Mo1—C11 angle as small as 76.04 (11)°.

In addition, an intra­molecular C—H⋯O inter­action [(C4)H4⋯O2^i^ = 2.60 Å, C4 —H4 ⋯O2^i^ = 123°] consolidates the mol­ecular conformation (Table 1 ▸; Fig. 2 ▸).

## 3. Supra­molecular features

**Figure 3:**
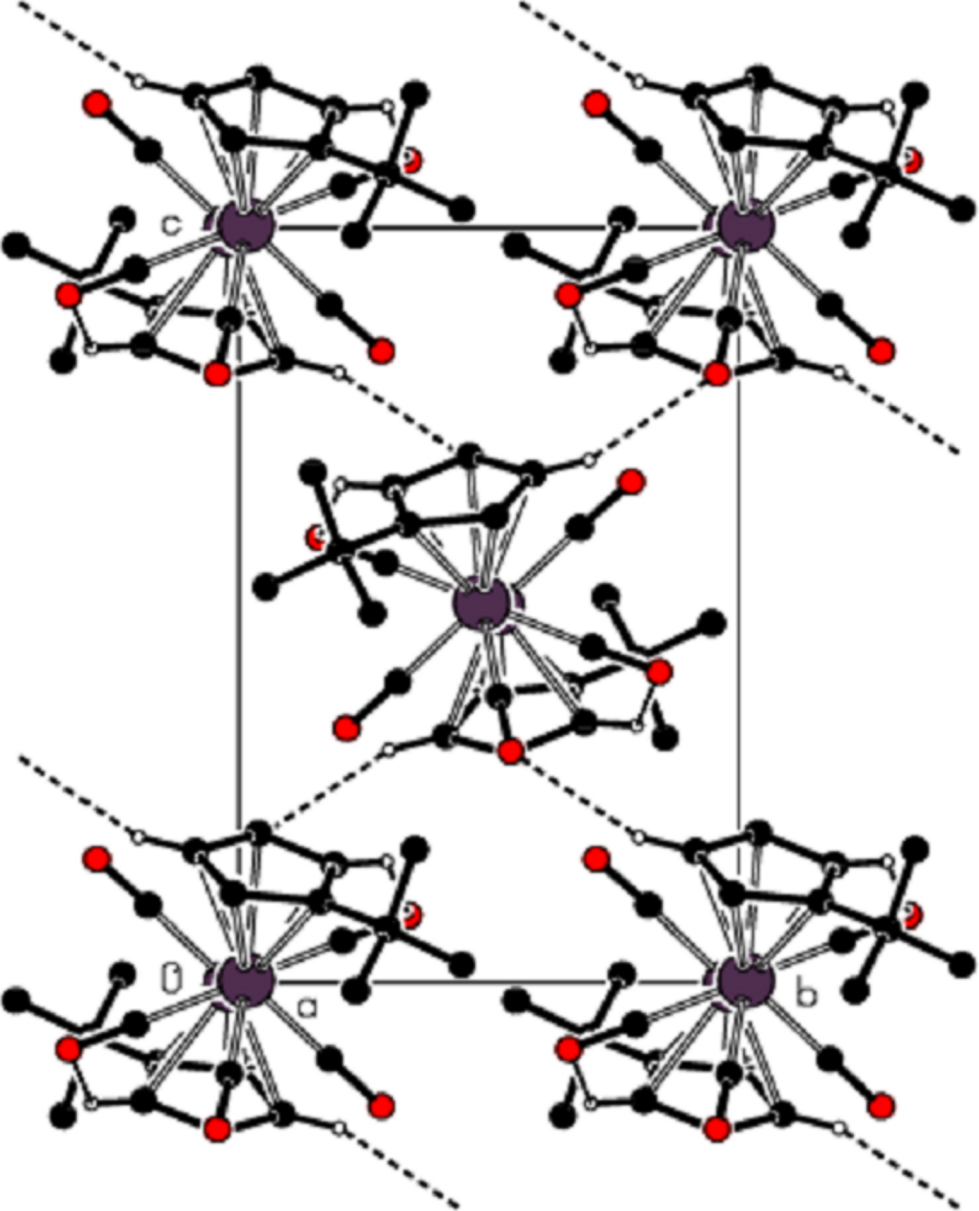
View of the non-classical hydrogen-bonding inter­actions down the *a* axis.

In the crystal of the title compound, an inter­molecular C—H⋯O inter­action [(C2)H2⋯O3^ii^ = 2.52 Å, C2—H2⋯O3^ii^ = 160°; symmetry code: (ii) *x*, −*y* + 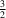, *z* + 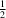] is important and close to linear (Table 1 ▸; Figs. 2 ▸–5 ▸ ▸ ▸). These inter­actions connect the mol­ecules into layers parallel to the *bc* plane (Figs. 3 ▸–5 ▸ ▸).

## 4. Database survey

A survey of the Cambridge Structural Database (CSD, Version 5.43, last update November 2022; Groom *et al.*, 2016 ▸) returned 25 hits for a search with the bis­[(η^5^-cyclo­penta­dien­yl)tri­carbonyl­molybdenum] moiety as the search criterion. The four closest similar compounds are those with refcodes CYPMOC01 (Gould *et al.*, 1988 ▸), CYPMOC10 (Adams *et al.*, 1974 ▸), GAKVUJ (Clegg *et al.*, 1988 ▸), and TIVLAL (Hughes *et al.*, 1996 ▸).

CYPMOC10 crystallizes in the monoclinic *P*2_1_/*c* space group with *Z* = 2, CYPMOC10 and GAKVUJ crystallize in the monoclinic *P*2_1_/*n* space group with *Z* = 2, while CYPMOC01 crystallizes in the monoclinic *I*2 space group with *Z* = 2, and TIVLAL in the triclinic *P*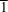 space group with *Z* = 1.

Although the Mo—Mo distances in these structures vary slightly depending on the steric effects caused by the groups attached to the Cp rings, the values may be compared within the error limits of the experiments. The Mo—Mo distances are 3.2239 (11) Å for CYPMOC01, 3.235 (1) Å for CYPMOC10, 3.281 (1) Å for GAKVUJ, and 3.253 (1) Å for TIVLAL. The average length of the Mo—Mo bond in these structures is 3.263 (8) Å, which is in agreement with the length of the Mo—Mo bond for the title compound [3.2323 (3) Å]. In all these structures, the Mo—C≡O angles deviate only slightly from linearity due to the steric effects mentioned.

## 5. Synthesis and crystallization

The binuclear complex [Mo(^*t*^BuCp)(CO)_3_]_2_ was synthesized according to a reported protocol (Manning *et al.*, 1990 ▸). Under an inert atmosphere, 195 mg (5 mmol) of sodium amide and 0.7 ml (5 mmol) of freshly distilled *tert*-butyl­cyclo­penta­diene in diglyme (100 ml) were heated for 3 h at 318–323 K. After the mixture had cooled to room temperature, 1.32 g (5 mmol) of molybdenum hexa­carbonyl were added and the mixture heated at 423 K for 40 min. The yellow-colored reaction mixture was cooled to room temperature and 40 g of iron(III) sulfate [Fe_2_(SO_4_)_3_·9H_2_O] in 400 ml of water and 25 ml of glacial acetic acid were added. The reaction mixture turned red, and crystals precipitated from it, which were filtered and further washed with water, methanol and pentane. After drying, 2.56 g (85%) of a dark-red crystalline solid of the title compound were obtained. Melting point: 441–442 K (with decomposition); ^1^H NMR, 300 MHz, (CD_2_Cl_2_), δ(p.p.m.): 1.19 (*s*, 18H, 6CH_3_), 5.02 (*s*, 4H, α-CH), 5.20 (*s*, 4H, β-CH); IR: *ν*(cm^−1^): 1948, 1912 and 1858 (C≡O). Deep-red crystals of the title complex suitable for single crystal X-ray analysis were grown in toluene at a temperature of 263 K.

## 6. Refinement

Crystal data, data collection and structure refinement details are summarized in Table 2 ▸. H atoms were included in calculated positions and treated as riding: C—H = 0.95–0.98 Å with *U*_iso_(H) = 1.5*U*_eq_(C-meth­yl) and 1.2*U*_eq_(C) for other H atoms.

## Supplementary Material

Crystal structure: contains datablock(s) I. DOI: 10.1107/S2056989024006959/wm5725sup1.cif

Structure factors: contains datablock(s) I. DOI: 10.1107/S2056989024006959/wm5725Isup2.hkl

CCDC reference: 2243119

Additional supporting information:  crystallographic information; 3D view; checkCIF report

## Figures and Tables

**Figure 4:**
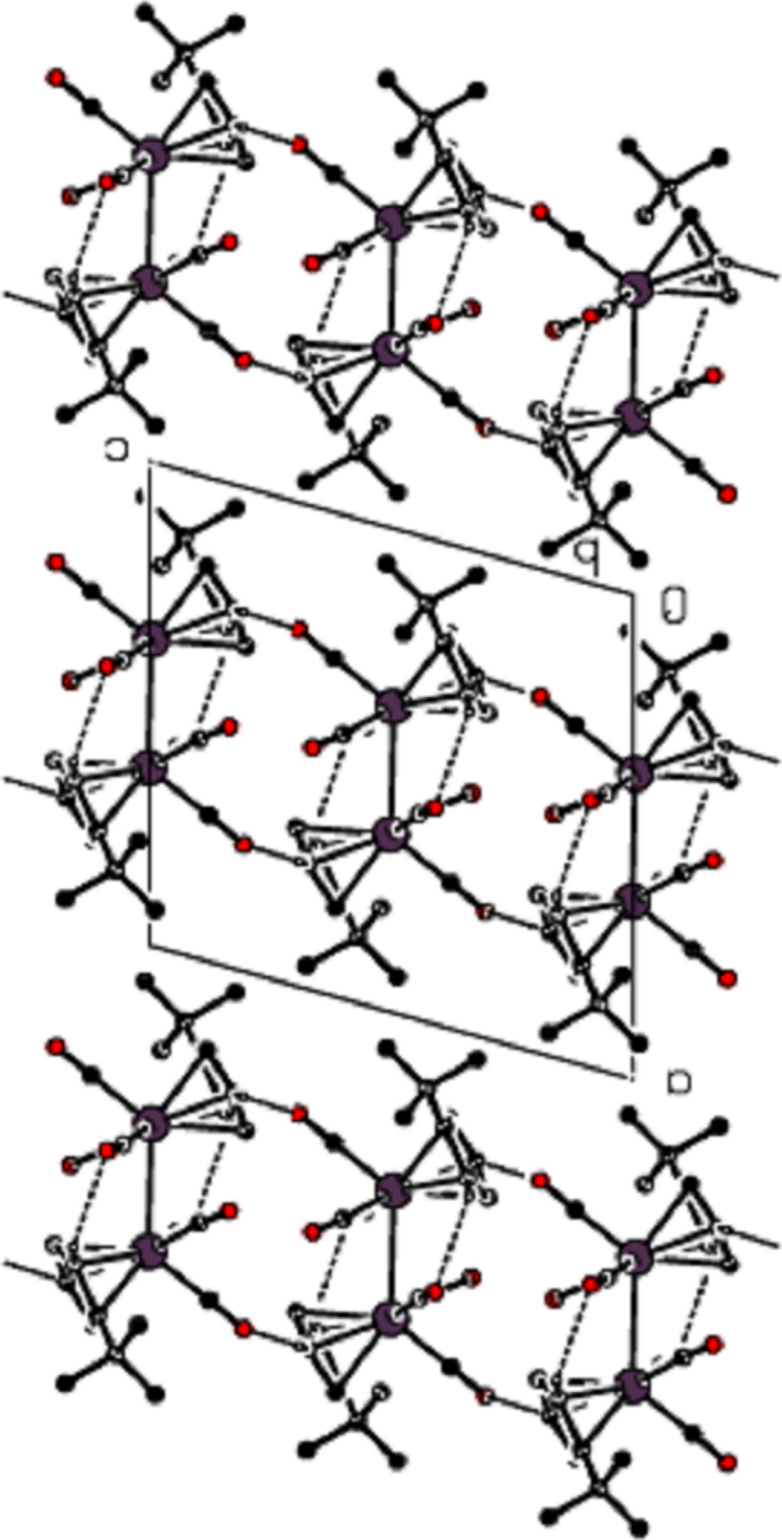
View of the non-classical hydrogen-bonding inter­actions down the *b* axis.

**Figure 5:**
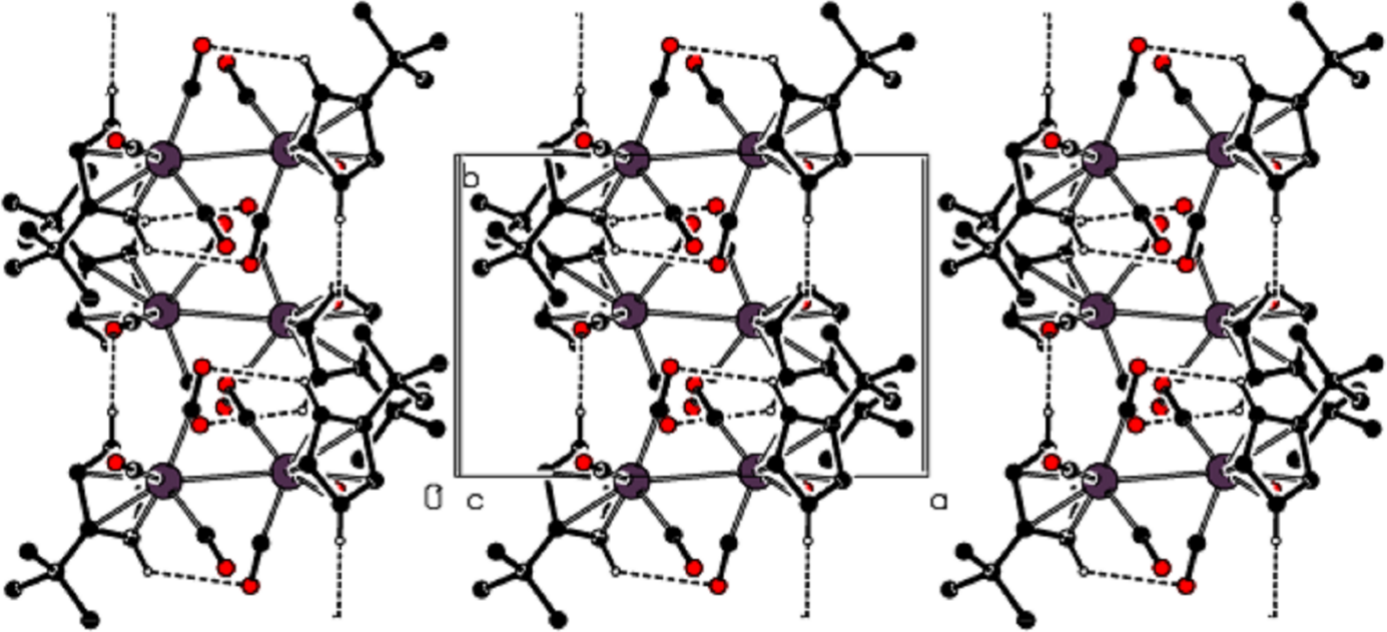
View of the non-classical hydrogen-bonding inter­actions down the *c* axis.

**Table 1:** Hydrogen-bond geometry (Å, °)

*D*—H⋯*A*	*D*—H	H⋯*A*	*D*⋯*A*	*D*—H⋯*A*
C4—H4⋯O2^i^	0.95	2.60	3.212 (3)	123
C2—H2⋯O3^ii^	0.95	2.52	3.429 (4)	160

Symmetry codes: (i) 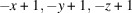; (ii) 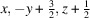.

**Table 2:** Experimental details

Crystal data	
Chemical formula	[Mo_2_(C_9_H_13_)_2_(CO)_6_]
*M*_r_	602.35
Crystal system, space group	Monoclinic, *P*2_1_/*c*
Temperature (K)	150
*a*, *b*, *c* (Å)	12.1857 (7), 8.0515 (5), 12.6153 (8)
β (°)	105.490 (2)
*V* (Å^3^)	1192.77 (13)
*Z*	2
Radiation type	Mo *K*α
μ (mm^−1^)	1.09
Crystal size (mm)	0.34 × 0.27 × 0.14

Data collection	
Diffractometer	Bruker D8 Quest PHOTON 100 detector
Absorption correction	Multi-scan (*SADABS*; Krause *et al.*, 2015 ▸)
*T*_min_, *T*_max_	0.692, 0.845
No. of measured, independent and observed [*I* > 2σ(*I*)] reflections	13856, 2269, 1892
*R*_int_	0.051
(sin θ/λ)_max_ (Å^−1^)	0.611

Refinement	
*R*[*F*^2^ > 2σ(*F*^2^)], *wR*(*F*^2^), *S*	0.025, 0.054, 1.03
No. of reflections	2269
No. of parameters	148
H-atom treatment	H-atom parameters constrained
Δρ_max_, Δρ_min_ (e Å^−3^)	0.38, −0.34

Computer programs: *APEX4* and *SAINT* (Bruker, 2018 ▸), *SHELXT* (Sheldrick, 2015*a* ▸), *SHELXL2018* (Sheldrick, 2015*b* ▸), *ORTEP-3 for Windows* (Farrugia, 2012 ▸) and *PLATON* (Spek, 2020 ▸).

## supplementary crystallographic information

### Bis[(η5-tert-butylcyclopentadienyl)tricarbonylmolybdenum(I)](Mo—Mo) Crystal data

**Table:**

[Mo_2_(C_9_H_13_)_2_(CO)_6_]	*F*(000) = 604
*M**_r_* = 602.35	*D*_x_ = 1.677 Mg m^−^^3^
Monoclinic, *P*2_1_/*c*	Mo *K*α radiation, λ = 0.71073 Å
*a* = 12.1857 (7) Å	Cell parameters from 3077 reflections
*b* = 8.0515 (5) Å	θ = 3.0–23.6°
*c* = 12.6153 (8) Å	µ = 1.09 mm^−^^1^
β = 105.490 (2)°	*T* = 150 K
*V* = 1192.77 (13) Å^3^	Prism, red
*Z* = 2	0.34 × 0.27 × 0.14 mm

### Bis[(η5-tert-butylcyclopentadienyl)tricarbonylmolybdenum(I)](Mo—Mo) Data collection

**Table:**

Bruker D8 Quest PHOTON 100 detector diffractometer	1892 reflections with *I* > 2σ(*I*)
φ and ω scans	*R*_int_ = 0.051
Absorption correction: multi-scan (SADABS; Krause *et al.*, 2015)	θ_max_ = 25.7°, θ_min_ = 1.7°
*T*_min_ = 0.692, *T*_max_ = 0.845	*h* = −11→14
13856 measured reflections	*k* = −8→9
2269 independent reflections	*l* = −15→12

### Bis[(η5-tert-butylcyclopentadienyl)tricarbonylmolybdenum(I)](Mo—Mo) Refinement

**Table:**

Refinement on *F*^2^	0 restraints
Least-squares matrix: full	Hydrogen site location: inferred from neighbouring sites
*R*[*F*^2^ > 2σ(*F*^2^)] = 0.025	H-atom parameters constrained
*wR*(*F*^2^) = 0.054	*w* = 1/[σ^2^(*F*_o_^2^) + (0.0188*P*)^2^ + 0.5962*P*] where *P* = (*F*_o_^2^ + 2*F*_c_^2^)/3
*S* = 1.03	(Δ/σ)_max_ = 0.001
2269 reflections	Δρ_max_ = 0.38 e Å^−^^3^
148 parameters	Δρ_min_ = −0.34 e Å^−^^3^

### Bis[(η5-tert-butylcyclopentadienyl)tricarbonylmolybdenum(I)](Mo—Mo) Special details

**Table:**

Geometry. All esds (except the esd in the dihedral angle between two l.s. planes) are estimated using the full covariance matrix. The cell esds are taken into account individually in the estimation of esds in distances, angles and torsion angles; correlations between esds in cell parameters are only used when they are defined by crystal symmetry. An approximate (isotropic) treatment of cell esds is used for estimating esds involving l.s. planes.

### Bis[(η5-tert-butylcyclopentadienyl)tricarbonylmolybdenum(I)](Mo—Mo) Fractional atomic coordinates and isotropic or equivalent isotropic displacement parameters (Å^2^)

**Table:**

	*x*	*y*	*z*	*U*_iso_*/*U*_eq_	
C1	0.8160 (2)	0.5120 (3)	0.6172 (2)	0.0197 (6)	
H1	0.871623	0.568453	0.590368	0.024*	
C2	0.7428 (2)	0.5865 (4)	0.6739 (2)	0.0224 (7)	
H2	0.741395	0.700674	0.692500	0.027*	
C3	0.6726 (2)	0.4613 (3)	0.6979 (2)	0.0206 (6)	
H3	0.615467	0.476238	0.735605	0.025*	
C4	0.7017 (2)	0.3089 (3)	0.6559 (2)	0.0184 (6)	
H4	0.666266	0.204813	0.659823	0.022*	
C5	0.7923 (2)	0.3371 (3)	0.6071 (2)	0.0172 (6)	
C6	0.8635 (2)	0.2032 (3)	0.5714 (2)	0.0230 (7)	
C7	0.9527 (3)	0.1482 (4)	0.6762 (3)	0.0381 (9)	
H7A	1.000785	0.243025	0.707718	0.057*	
H7B	1.000107	0.060012	0.658058	0.057*	
H7C	0.914114	0.106311	0.729745	0.057*	
C8	0.7904 (3)	0.0537 (4)	0.5238 (3)	0.0491 (11)	
H8A	0.838440	−0.032870	0.504889	0.074*	
H8B	0.732053	0.087341	0.457472	0.074*	
H8C	0.753562	0.010529	0.578296	0.074*	
C9	0.9232 (4)	0.2666 (5)	0.4893 (3)	0.0625 (13)	
H9A	0.866552	0.299405	0.421602	0.094*	
H9B	0.971821	0.178851	0.472642	0.094*	
H9C	0.970149	0.362890	0.520100	0.094*	
C10	0.5452 (2)	0.3181 (3)	0.3980 (2)	0.0179 (6)	
C11	0.5746 (2)	0.7073 (4)	0.4444 (2)	0.0189 (6)	
C12	0.6983 (2)	0.5190 (3)	0.3786 (2)	0.0197 (6)	
O1	0.50217 (16)	0.2156 (2)	0.33646 (16)	0.0251 (5)	
O2	0.55213 (16)	0.8396 (2)	0.41126 (16)	0.0259 (5)	
O3	0.73768 (18)	0.5429 (2)	0.30634 (17)	0.0302 (5)	
Mo1	0.63311 (2)	0.48528 (3)	0.50317 (2)	0.01290 (8)	

### Bis[(η5-tert-butylcyclopentadienyl)tricarbonylmolybdenum(I)](Mo—Mo) Atomic displacement parameters (Å^2^)

**Table:**

	*U*^11^	*U*^22^	*U*^33^	*U*^12^	*U*^13^	*U*^23^
C1	0.0147 (15)	0.0257 (16)	0.0162 (14)	−0.0030 (12)	−0.0001 (11)	0.0002 (12)
C2	0.0230 (18)	0.0223 (15)	0.0169 (16)	0.0053 (13)	−0.0031 (13)	−0.0044 (12)
C3	0.0170 (16)	0.0344 (17)	0.0091 (14)	0.0089 (13)	0.0014 (11)	0.0011 (12)
C4	0.0162 (16)	0.0218 (15)	0.0156 (15)	0.0028 (12)	0.0016 (12)	0.0067 (12)
C5	0.0129 (16)	0.0212 (15)	0.0145 (15)	0.0022 (11)	−0.0015 (12)	0.0012 (11)
C6	0.0204 (17)	0.0250 (16)	0.0218 (16)	0.0099 (13)	0.0026 (13)	0.0012 (12)
C7	0.030 (2)	0.042 (2)	0.035 (2)	0.0190 (16)	−0.0021 (16)	−0.0046 (16)
C8	0.036 (2)	0.040 (2)	0.065 (3)	0.0123 (17)	0.0026 (19)	−0.0283 (19)
C9	0.079 (3)	0.054 (3)	0.077 (3)	0.040 (2)	0.061 (3)	0.029 (2)
C10	0.0174 (16)	0.0190 (15)	0.0184 (15)	0.0064 (12)	0.0064 (12)	0.0050 (12)
C11	0.0124 (16)	0.0267 (17)	0.0170 (15)	−0.0024 (12)	0.0030 (12)	−0.0021 (12)
C12	0.0197 (16)	0.0155 (15)	0.0230 (16)	0.0041 (12)	0.0039 (13)	0.0011 (12)
O1	0.0268 (12)	0.0224 (11)	0.0241 (12)	0.0005 (9)	0.0032 (9)	−0.0097 (9)
O2	0.0257 (12)	0.0156 (11)	0.0355 (13)	0.0020 (9)	0.0067 (10)	0.0072 (9)
O3	0.0413 (14)	0.0292 (12)	0.0274 (12)	0.0044 (10)	0.0216 (11)	0.0055 (9)
Mo1	0.01234 (14)	0.01388 (13)	0.01223 (13)	0.00102 (10)	0.00287 (9)	0.00043 (10)

### Bis[(η5-tert-butylcyclopentadienyl)tricarbonylmolybdenum(I)](Mo—Mo) Geometric parameters (Å, º)

**Table:**

C1—C2	1.418 (4)	C6—C7	1.536 (4)
C1—C5	1.437 (4)	C7—H7A	0.9800
C1—Mo1	2.318 (3)	C7—H7B	0.9800
C1—H1	0.9500	C7—H7C	0.9800
C2—C3	1.406 (4)	C8—H8A	0.9800
C2—Mo1	2.357 (3)	C8—H8B	0.9800
C2—H2	0.9500	C8—H8C	0.9800
C3—C4	1.419 (4)	C9—H9A	0.9800
C3—Mo1	2.381 (3)	C9—H9B	0.9800
C3—H3	0.9500	C9—H9C	0.9800
C4—C5	1.419 (4)	C10—O1	1.158 (3)
C4—Mo1	2.360 (3)	C10—Mo1	1.990 (3)
C4—H4	0.9500	C11—O2	1.151 (3)
C5—C6	1.525 (4)	C11—Mo1	1.992 (3)
C5—Mo1	2.356 (3)	C12—O3	1.154 (3)
C6—C9	1.505 (4)	C12—Mo1	1.960 (3)
C6—C8	1.522 (4)		
			
C2—C1—C5	108.7 (2)	C6—C8—H8B	109.5
C2—C1—Mo1	73.84 (15)	H8A—C8—H8B	109.5
C5—C1—Mo1	73.53 (15)	C6—C8—H8C	109.5
C2—C1—H1	125.6	H8A—C8—H8C	109.5
C5—C1—H1	125.6	H8B—C8—H8C	109.5
Mo1—C1—H1	118.8	C6—C9—H9A	109.5
C3—C2—C1	107.8 (2)	C6—C9—H9B	109.5
C3—C2—Mo1	73.69 (15)	H9A—C9—H9B	109.5
C1—C2—Mo1	70.87 (15)	C6—C9—H9C	109.5
C3—C2—H2	126.1	H9A—C9—H9C	109.5
C1—C2—H2	126.1	H9B—C9—H9C	109.5
Mo1—C2—H2	121.1	O1—C10—Mo1	174.6 (2)
C2—C3—C4	108.2 (3)	O2—C11—Mo1	173.1 (2)
C2—C3—Mo1	71.79 (15)	O3—C12—Mo1	178.2 (2)
C4—C3—Mo1	71.76 (15)	C12—Mo1—C10	79.64 (11)
C2—C3—H3	125.9	C12—Mo1—C11	76.04 (11)
C4—C3—H3	125.9	C10—Mo1—C11	106.46 (11)
Mo1—C3—H3	122.2	C12—Mo1—C1	87.50 (11)
C3—C4—C5	108.9 (2)	C10—Mo1—C1	137.22 (10)
C3—C4—Mo1	73.42 (15)	C11—Mo1—C1	109.68 (10)
C5—C4—Mo1	72.33 (15)	C12—Mo1—C5	93.81 (10)
C3—C4—H4	125.5	C10—Mo1—C5	104.20 (10)
C5—C4—H4	125.5	C11—Mo1—C5	145.19 (10)
Mo1—C4—H4	120.4	C1—Mo1—C5	35.79 (9)
C4—C5—C1	106.2 (2)	C12—Mo1—C2	115.65 (11)
C4—C5—C6	125.8 (2)	C10—Mo1—C2	156.39 (10)
C1—C5—C6	127.1 (3)	C11—Mo1—C2	95.11 (10)
C4—C5—Mo1	72.64 (14)	C1—Mo1—C2	35.29 (9)
C1—C5—Mo1	70.68 (14)	C5—Mo1—C2	58.97 (9)
C6—C5—Mo1	129.98 (18)	C12—Mo1—C4	127.37 (10)
C9—C6—C8	109.7 (3)	C10—Mo1—C4	98.49 (10)
C9—C6—C5	112.4 (2)	C11—Mo1—C4	149.01 (10)
C8—C6—C5	110.9 (2)	C1—Mo1—C4	58.45 (10)
C9—C6—C7	109.3 (3)	C5—Mo1—C4	35.03 (9)
C8—C6—C7	108.5 (3)	C2—Mo1—C4	58.06 (10)
C5—C6—C7	106.1 (2)	C12—Mo1—C3	145.58 (11)
C6—C7—H7A	109.5	C10—Mo1—C3	123.87 (10)
C6—C7—H7B	109.5	C11—Mo1—C3	114.21 (10)
H7A—C7—H7B	109.5	C1—Mo1—C3	58.09 (10)
C6—C7—H7C	109.5	C5—Mo1—C3	58.36 (9)
H7A—C7—H7C	109.5	C2—Mo1—C3	34.52 (10)
H7B—C7—H7C	109.5	C4—Mo1—C3	34.82 (9)
C6—C8—H8A	109.5		
			
C5—C1—C2—C3	−0.9 (3)	C2—C1—C5—C4	1.6 (3)
Mo1—C1—C2—C3	64.91 (18)	Mo1—C1—C5—C4	−64.44 (17)
C5—C1—C2—Mo1	−65.81 (18)	C2—C1—C5—C6	−167.9 (2)
C1—C2—C3—C4	−0.1 (3)	Mo1—C1—C5—C6	126.0 (3)
Mo1—C2—C3—C4	62.92 (18)	C2—C1—C5—Mo1	66.01 (18)
C1—C2—C3—Mo1	−63.06 (18)	C4—C5—C6—C9	160.1 (3)
C2—C3—C4—C5	1.2 (3)	C1—C5—C6—C9	−32.3 (4)
Mo1—C3—C4—C5	64.09 (18)	Mo1—C5—C6—C9	63.0 (4)
C2—C3—C4—Mo1	−62.93 (19)	C4—C5—C6—C8	37.0 (4)
C3—C4—C5—C1	−1.7 (3)	C1—C5—C6—C8	−155.4 (3)
Mo1—C4—C5—C1	63.12 (17)	Mo1—C5—C6—C8	−60.1 (3)
C3—C4—C5—C6	168.0 (2)	C4—C5—C6—C7	−80.5 (3)
Mo1—C4—C5—C6	−127.2 (3)	C1—C5—C6—C7	87.0 (3)
C3—C4—C5—Mo1	−64.78 (18)	Mo1—C5—C6—C7	−177.7 (2)

### Bis[(η5-tert-butylcyclopentadienyl)tricarbonylmolybdenum(I)](Mo—Mo) Hydrogen-bond geometry (Å, º)

**Table:**

*D*—H···*A*	*D*—H	H···*A*	*D*···*A*	*D*—H···*A*
C4—H4···O2^i^	0.95	2.60	3.212 (3)	123
C2—H2···O3^ii^	0.95	2.52	3.429 (4)	160

Symmetry codes: (i) −*x*+1, −*y*+1, −*z*+1; (ii) *x*, −*y*+3/2, *z*+1/2.
